# Long Non-coding RNA PVT1 is Involved in the Pathological Mechanism of Pulpitis by Regulating miR-128-3p

**DOI:** 10.3290/j.ohpd.b3147193

**Published:** 2022-06-20

**Authors:** Lin Xia, Jian Wang, Yue Qi, Yongjie Fei, Dongmei Wang

**Affiliations:** a Attending Physician, Department of Stomatology, Baoshan Hospital of Integrated Traditional Chinese Medicine and Western Medicine, Shanghai, China. Data analysis, wrote the first draft of the manuscript, interpreted the results, critically revised the manuscript, read and approved the final manuscript.; b Associate Professor, Department of Stomatology, Dongying Hospital of Traditional Chinese Medicine, Dongying, China. Data analysis, wrote the first draft of the manuscript, read and approved the final manuscript.; c Attending Physician, Department of Stomatology, Dongying Hospital of Traditional Chinese Medicine, Dongying, China. Data analysis, wrote the first draft of the manuscript, read and approved the final manuscript.; d Attending Physician, Department of Stomatology, Dongying Hospital of Traditional Chinese Medicine, Dongying, China. Contributed to analysis planning, read and approved the final manuscript.; e Associate Professor, Department of Stomatology, Dongying Hospital of Traditional Chinese Medicine, Dongying, China. Idea, interpreted the results, critically revised the manuscript, read and approved the final manuscript.

**Keywords:** LPS, miR-128-3p, pulpitis, PVT1

## Abstract

**Purpose:**

Pulpitis is a common disease in stomatology, which is caused by dental pulp infection. It was found that long non-coding RNA regulates inflammation and repair responses through competitively sponging microRNAs. This study explored the expression and clinical significance of PVT1 in pulpitis patients, and further investigated the possible regulatory mechanism of PVT1 on pulpitis through in-vitro experiments.

**Materials and Methods:**

The expression of PVT1 and miR-128-3p was detected through RT-qPCR. An ROC curve was drawn to estimate the diagnostic significance of PVT1 and miR-128-3p for pulpitis. An in-vitro pulpitis cell model was constructed to evaluate the effects of PVT1 or miR-128-3p on cell proliferation, apoptosis, and inflammatory response. The Luciferase reporter gene explored the interaction between PVT1 and miR-128-3p.

**Results:**

The expression of PVT1 increased, while the miR-128-3p level decreased, in the saliva of pulpitis patients. ROC curves showed that both PVT1 and miR-128-3p had the potential to diagnose pulpitis. This in-vitro study revealed that the expression of PVT1 was increased in the pulpitis cell model. A low level of PVT1 suppressed the hDPCs injury induced by LPS. The Luciferase reporter gene verified the targeting relationship between PVT1 and miR-128-3p, and the latter was negatively regulated by PVT1. Further in-vitro studies showed that inhibition of miR-128-3p could reverse the effect of si-PVT1 on cell viability, cell apoptosis and inflammatory response.

**Conclusion:**

This study revealed that knockdown of PVT1 may suppress the damage in pulpitis cell models induced by LPS via targeting miR-128-3p.

Dental pulp is a connective tissue located in the center of teeth, surrounded by enamel and dentin, which is necessary for tooth immune function.^3,19^ Pulpitis is defined as inflammation that occurs when bacteria or toxins invade the pulp at the tooth center.^6^ Dental caries is the most common cause of pulpitis, and bacteria play a vital role in the occurrence of pulpitis.^1,9^ The most common symptom of pulpitis is severe tooth pain, and the main clinical therapy is endodontic treatment.^10^ Without proper and timely treatment, pulpitis may lead to pulp necrosis, and even severe oral cavity infections,^12^ which will eventually lead to a serious medical and economic burden.

Long non-coding RNA (lncRNA) is an RNA molecule over 200 nucleotides long. It does not encode protein, but regulates gene expression in the form of RNA at epigenetic, transcriptional, and post-transcriptional levels.^15^ It has been reported that various factors, including lncRNA and cytokines, are involved in the related processes of oral inflammatory diseases.^11^ For example, Liu et al^14^ detected by RNAscope technology that the expression of maternally expressed gene 3 (MEG3) in human inflammatory pulp tissues was stronger than that in human normal dental pulp tissues. MALAT1, acting as an miR-20a sponge, induces Toll-like receptor 4 (TLR4) signal transduction, leading to inflammatory reaction of human gingival fibroblasts (HGFs).^13^ Plasmacytoma variant 1 (PVT1), located on human chromosome 8q24, was recently found to be involved in oral inflammatory diseases.^17^ Lei et al^12^ found that the PVT1 leve l was increased markedly in pulp tissues of pulpitis patients.^12^ However, there is still relatively little research on the role of PVT1 in the pathogenesis of pulpitis. Studies have found that lncRNA usually interacts with microRNAs (miRNAs) as competitive endogenous RNAs and participates in the regulation of target genes.^4^ miRNA is a small non-coding RNA with a length of about 20 nucleotides. It has been reported that miR-128-3p is abnormally expressed in some inflammatory diseases, such as sepsis and atherosclerosis.^8,23^ Exploring whether miR-128-3p is involved in the regulation of PVT1 on pulpitis is interesting and meaningful for us to further understand the mechanism of PVT1 participating in pulpitis.

In the present study, PVT1 was found to be elevated in saliva of pulpitis patients, and the PVT1 knockdown restrained the production of inflammatory factors. Additionally, our study further investigated the relationship between PVT1 and miR-128-3p, and we speculated that PVT1 was involved in the development of pulpitis by targeted regulating miR-128-3p.

## Materials and Methods

### Patients and Samples

This research protocol was completed in Dongying Hospital of Traditional Chinese Medicine from October 2019 to February 2021. The present study recruited 178 participants, of whom 93 patients with pulpitis made up the case group and 85 healthy people without oral diseases comprised the healthy control group. The age, gender, BMI, smoking and drinking history of subjects in the control group were matched with those in the case group. Subjects who took NSAIDs and oral antibiotics and had a history of smoking and drinking during the two weeks prior to study initiation were excluded. Saliva from all subjects was collected in sterile tubes and stored in a freezer at -80°C for subsequent study. This study protocol was implemented after being approved by the Ethics Committee of Dongying Hospital of Traditional Chinese Medicine, and all subjects signed written informed consent.

### RNA Extraction and RT-qPCR

All RNA was obtained by TRIzol kit (Invitrogen; Carlsbad, CA, USA). All RNA of PVT1 and miR-128-3p was reverse transcribed into cDNA by using Super-Script II reverse transcriptase kit (Invitrogen) and miScript reverse transcription kit (Qiagen; Hilden, Germany). Subsequently, RT-qPCR analysis was carried out on the 7300 Real-Time PCR System (Applied Biosystems; Foster City, CA, USA) according to the SYBR Green PCR Kit (Qiagen) instructions. U6 and β-actin were arranged as the internal references for miR-128-3p and PVT1, respectively. The relative expression of PVT1 and miR-128-3p was calculated and normalised to β-actin and U6 using the 2–ΔΔCt method.

### Cell Culture and LPS Induction

Human dental pulp cells (hDPCs) were supplied by the Shanghai Institute of Biochemistry and Cell Biology (Shanghai, China) and were cultivated in DMEM containing 10% FBS and 1% penicillin-streptomycin. hDPCs were cultured in an incubator composed of 5% CO_2_ and 95% air at 37°C. The construction of an in-vitro pulpitis cell model was based on a previously published article.^22^ In short, hDPCs were seeded into a 12-well plate and cultured overnight. Subsequently, hDPCs were exposed to *Escherichia coli* LPS (Sigma; St Louis, MO, USA) at concentrations of 0, 50, 100, 200 ng/ml for 24 h.

### Cell Transfection

si-NC/PVT1, mimic/inhibitor-NC, and miR-128-3p mimic/inhibitor needed for cell transfection were all provided by GenePharma (Suzhou; Zhejiang, China). Briefly, hDPCs were transfected with the biomacromolecules just mentioned via Lipofectamine 2000 (Invitrogen) in accordance with manufacturer’s instructions. After 24 h of cell transfection, LPS at a concentration of 200 ng/ml was added to hDPCs for further culture for 24 h.

### Cell Viability Assay

A cell counting kit-8 (CCK-8) assay was performed for the evaluation of cell viability. In brief, hDPCs were seeded into a 96-well plate at a density of 5x10^3^ cells/well. At 0, 24, 48 and 72 h after cell transfection and LPS induction, CCK-8 solution was added, and the cells were incubated in the dark for 2 h. The OD value at 450 nm was measured by a Microplate Reader (Thermo Scientific; Waltham, MA, USA).

### Cell Apoptosis Assay

Flow cytometry and an apoptosis detection kit (Invitrogen) were used to evaluate cell apoptosis. Firstly, hDPCs were inoculated in a 6-well plate at a density of 2 x 10^5^ cells/well. Then, the cells were collected and washed following the respective experimental treatment. The cells were resuspended with binding buffer, and 10 μl of annexin V-FITC solution and 5 μl of PI solution were added to the cell suspensions, respectively. Finally, after incubation in the dark for 15 min, 300 μl of binding buffer was added and analysed using flow cytometry.

### Enzyme-linked Immunosorbent Assay (ELISA)

After the cells were treated according to the prescribed experimental procedures, the total amount of TNF-α, IL-6, and IL-1β in cell supernatant was determined by ELISA kit (R&D; Minneapolis, MN, USA) according to the product specifications. The amount of protein was calculated based on the standard curve.

### Lactate Dehydrogenase (LDH) Release Detection Assay

LDH can be released from dead cells into the cell culture medium. The release of LDH was assessed using an LDH Cytotoxicity Detection Kit (Roche; Mannheim, Germany). In short, hDPCs were inoculated in a 24-well plate at a density of 2.5 x 10^4^ cells/well. After cell transfection and LPS induction, cell supernatant was collected and incubated with LDH reaction mixture for 30 min. Finally, the absorbance at 490 nm was read using a microplate reader.

### Luciferase Reporter Gene Assay

It was predicted that PVT1 has a complementary binding site to miR-128-3p. This was subsequently verified by luciferase reporter gene. Briefly, the 3’UTR of PVT1 containing miR-128-3p binding site was synthesised, and PVT1 3’UTR wild type (WT) plasmid and mutant (MUT) plasmid were constructed. hDPCs were co-transfected with the above plasmids and mimic/inhibitor-NC, miR-128-3p mimic/inhibitor. After 48 h of cell transfection, the luciferase activity of each group was analysed by a dual-luciferase reporter system (Promega; Madison, WI, USA).

### Data Analysis

Data analysis was performed using GraphPad Prism 7.0 (San Diego, CA, USA) and SPSS 22.0 (Armonk, NY, USA). Student’s t-test and one-way ANOVA followed by Tukey’s post-hoc test were used for intergroup and multigroup comparison, respectively. p < 0.05 was considered statistically significant. Data are shown as mean ± SD (standard deviation). Each experiment was repeated at least three times in parallel.

## Results

### Expression Level of Saliva PVT1 and miR-128-3p in Pulpitis Patients

**Fig 1:**
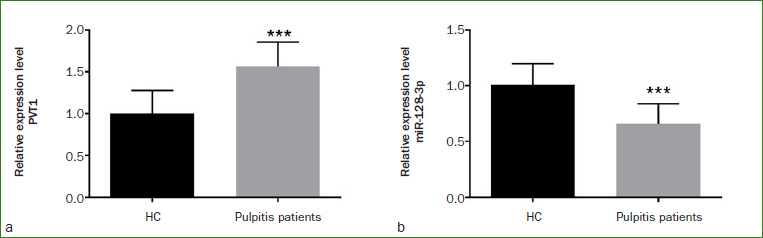
The gene expression levels were detected by RT-qPCR. The expression levels of a) PVT1 and b) miR-128-3p in saliva of all subjects were increased and decreased, respectively. ***p < 0.001.

The gene levels were analysed by RT-qPCR. As illustrated in Fig 1a, the PVT1 level was augmented in pulpitis patients compared with healthy controls (p < 0.001), while the level of miR-128-3p showed the opposite trend, that is, the level of miR-128-3p in pulpitis patients diminished statistically significantly (Fig 1b, p < 0.001).

### Diagnostic Value Analysis of PVT1 and miR-128-3p in Pulpitis

**Fig 2:**
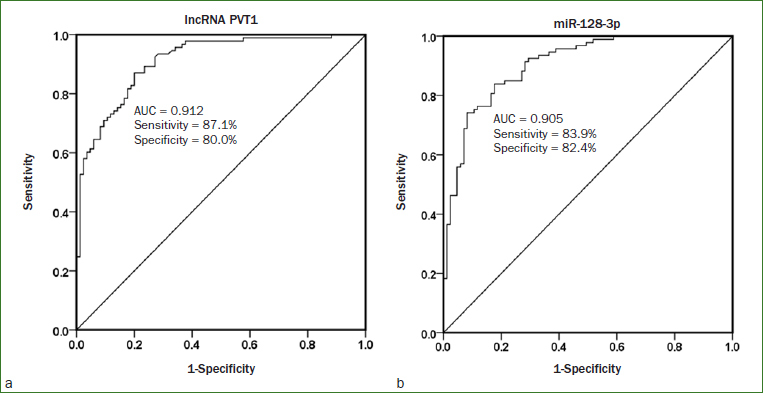
ROC analysis was used to evaluate the clinical diagnostic value of PVT1 and miR-128-3p for pulpitis. ROC curve showed that a) PVT1 and b) miR-128-3p had high clinical diagnostic accuracy in pulpitis.

ROC curves were constructed to assess the diagnostic significance of PVT1 and miR-128-3p for pulpitis. It was showed that PVT1 levels could be used to distinguish pulpitis patients from healthy controls. The AUC value was 0.912, with 87.1% sensitivity and 80.0% specificity (Fig 2a). Furthermore, the curve of miR-128-3p also exhibited good diagnostic performance, with an AUC value of 0.905, and sensitivity and specificity of 83.9% and 82.4%, respectively (Fig 2b).

### Effects of PVT1 on Cellular Function of LPS-stimulated hDPCs

**Fig 3:**
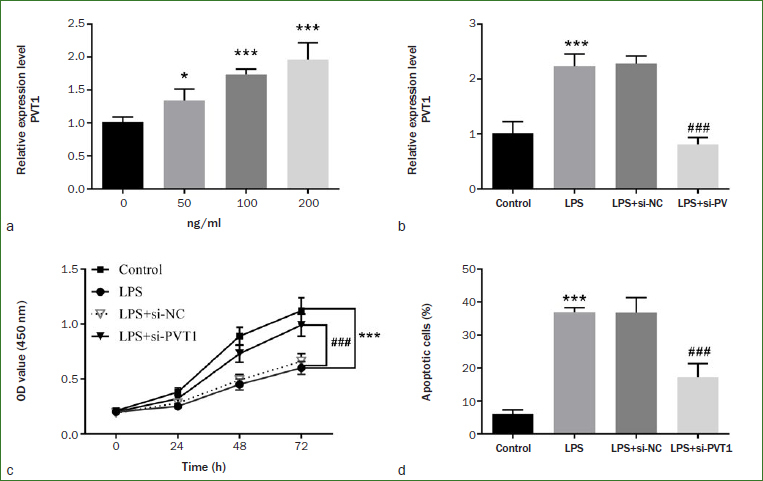
Overexpression of PVT1 inhibits cell proliferation and promotes cell apoptosis. a) PVT1 levels enhanced with the increase of LPS concentration. b) Transfection of si-PVT1 down-regulated LPS-induced PVT1 elevation. Effect of PVT1 on LPS-induced c) cell viability and d) cell apoptosis. ***p < 0.001, *p < 0.05, ###p < 0.001.

An in-vitro pulpitis cell model was established by LPS induction. LPS and si-PVT1 were allowed to treat hDPCs together to investigate whether LPS could affect cell function by regulating the expression of PVT1. As shown in Fig 3a, the level of intracellular PVT1 rose with the increase of LPS concentration (p < 0.001). Nevertheless, there was no statistically significant difference between the expression level of PVT1 at 200 ng/ml and at 100 ng/ml, so the experimental LPS concentration of 100 ng/ml was chosen. Figure 3b revealed that LPS-induced PVT1 elevation can be inhibited by transfection with si-PVT1 (p < 0.001). In addition, it could be seen that down-regulation of the PVT1 level reversed the decrease of cell viability and the increase of cell apoptosis induced by LPS (Figs 3c and 3d, p < 0.001).

### Effects of PVT1 on Cytokines of LPS-stimulated hDPCs

**Fig 4:**
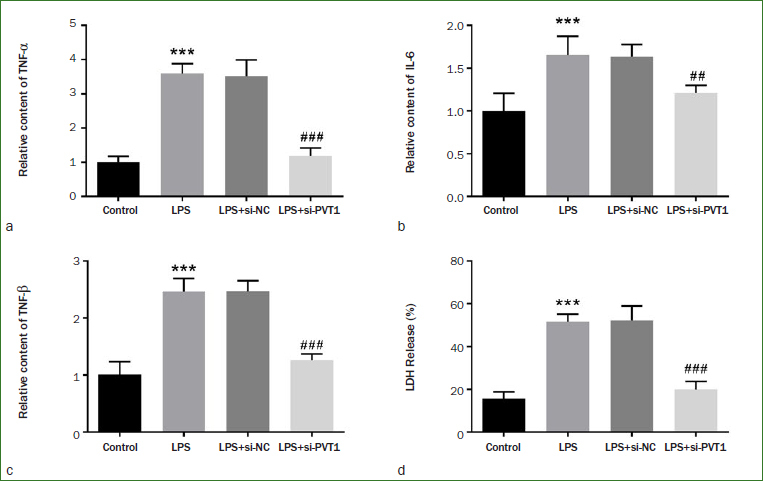
Overexpression of PVT1 promotes the inflammatory response in hDPCs. The levels of a) TNF-α, b) IL-6, and c) IL-1β were increased in LPS-induced hDPCs and were decreased after transfection of si-PVT1. d) The LPS-induced increase in LDH release was counteracted by PVT1 silencing. ***p < 0.001, ##p < 0.01, ###p < 0.001.

The levels of inflammatory factors were measured by ELISA. As can be seen from the results in Figs 4a to 4C, TNF-α, IL-1β, and IL-6 levels showed a statistically significantly increase after LPS exposure, indicating that LPS induced the inflammatory response in hDPCs (p < 0.001). Meanwhile, down-regulation of PVT1 correspondingly reduced the production of inflammatory factors (p < 0.01). Furthermore, LPS exposure increased the release of LDH, indicating that LPS exposure augmented the number of dead cells. However, transfection of si-PVT1 partially attenuated LPS-triggered LDH release (Fig 4d, p < 0.001).

### PVT1 Negatively Regulates the Expression Level of miR-128-3p

**Fig 5:**
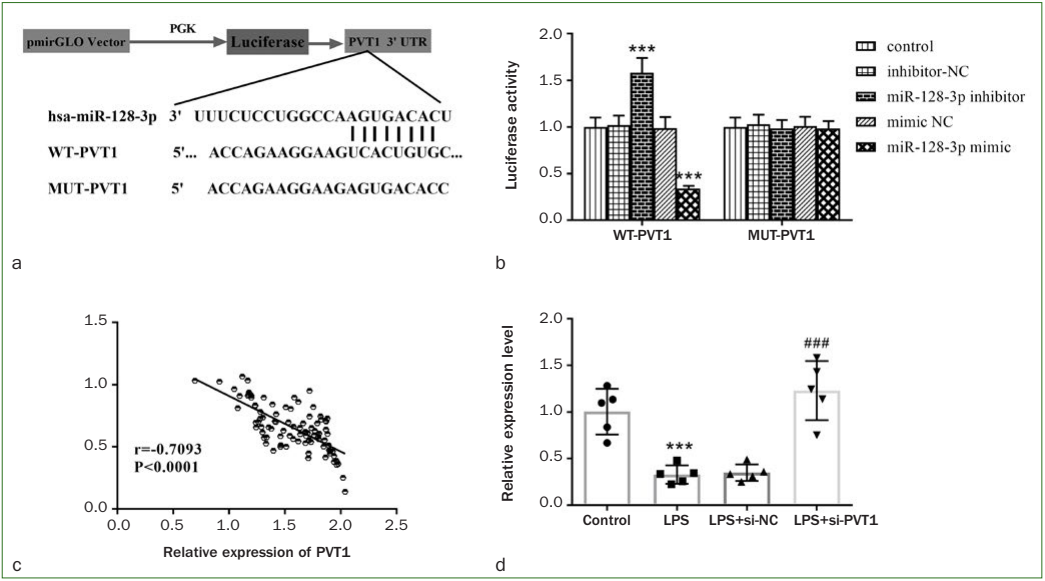
The targeting relationship between PVT1 and miR-128-3p in hDPCs was confirmed, and miR-128-3p was negatively regulated by PVT1. a) The complementary sites of PVT1 and miR-128-3p. b) Luciferase reporter gene assay confirmed the target relationship between PVT1 and miR-128-3p. c) Pearson correlation coefficient revealed that miR -128-3p was negatively regulated by PVT1. d) miR-128-3p level was increased after transfection of si-PVT1. ***p < 0.001, ###p < 0.001.

Through the bioinformatics software StarBase (Qu Lianghu research team, RNA Information Center, Sun Yat-Sen University, China; http://starbase.sysu.edu.cn), we predicted the target relationship in PVT1 and miR-128-3p; their complementary sequences are shown in Fig 5a. Luciferase reporter gene results showed that miR-128-3p inhibitor transfection dramatically diminished the luciferase activity in the WT-PVT1 group compared with that in MUT-PVT1 group, while miR-128-3p mimic transfection in the WT-PVT1 group showed the opposite, suggesting the direct combination effect of PVT1 and miR-128-3p in hDPCs (Fig 5b, p < 0.001). Moreover, Pearson correlation coefficient analysis showed that the miR-128-3p level was statistically significantly negatively correlated with PVT1 (Fig 5c, p < 0.001). We also found that miR-128-3p levels were diminished in LPS-induced hDPCs in comparison to the control group, while miR-128-3p levels were distinctly increased after PVT1 knockdown (Fig 5d, p < 0.001). In a word, PVT1 and miR-128-3p are the target relationship, and the latter is negatively regulated by PVT1 in hDPCs.

### Effects of miR-128-3p on Cell Function and Cytokines of LPS-stimulated hDPCs

**Fig 6:**
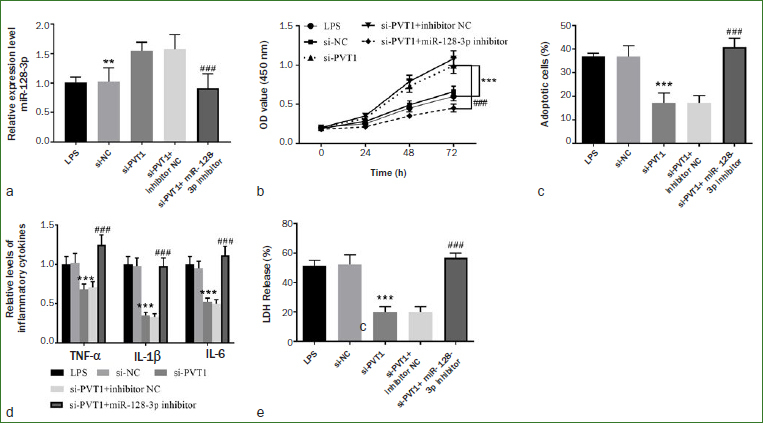
Knockdown of miR-128-3p inhibits cell proliferation and promotes apoptosis and inflammatory response. a) Silencing of PVT1 up-regulated the expression of miR-128-3p. Inhibition of miR-128-3p expression suppressed the b) cell viability and promoted the c) cell apoptosis. d) Silence of PVT1 reduced the expression levels of inflammatory factors, while inhibition of miR-128-3p enhanced the generation of inflammatory factors. e) Silence of PVT1 decreased the release of LDH, while suppression of miR-128-3p triggered the LDH release. ***p < 0.001, **p < 0.01, ###p < 0.001.

In light of the above results, hDPCs were co-transfected with si-PVT1 and miR-128-3p inhibitor to evaluate whether PVT1 is involved in cell function and generation of cytokines by regulating the expression of miR-128-3p. Figure 6a showed that PVT1 knockdown augmented the expression level of miR-128-3p, and similarly, miR-128-3p inhibitor reversed the above effects (p < 0.001). In-vitro cell experiments showed that cell viability and apoptosis were improved after silencing PVT1 expression, which manifested as increased cell viability increased and decreased cell apoptosis. However, these effects could be offset by transfection of miR-128-3p inhibitor (Figs 6b and 6c, p < 0.001). The same trend is also manifested in the regulation of inflammatory factors and LDH. As presented in Fig 6d and 6e, in LPS-induced hDPCs, the levels of cytokines and LDH decreased with the silence of PVT1, while the inhibition of miR-128-3p further upregulated the levels of cytokines and LDH, revealing that silence of PVT1 plays a part in regulation of cell functions and cytokines by improving miR-128-3p level.

## Discussion

Pulpitis is an inflammatory reaction in dental pulp tissue, mainly caused by bacterial infection.^20^ At present, a large number of studies have showed that lncRNA is related to the pathological mechanism of oral inflammatory diseases, including pulpitis, periodontitis, and periapical periodontitis.^18^ In this study, we found that the level of PVT1 in saliva of patients with pulpitis increased, while the expression of miR-128-3p decreased. ROC curve analysis showed that both PVT1 and miR-128-3p exhibited clinical diagnostic value for pulpitis. We studied the molecular mechanism of LPS-induced hDPCs injury through in vitro cell experiments. LPS stimulation can significantly upregulate the level of PVT1 in hDPCs, and at the same time, the inflammatory response of hDPCs was activated, and cell viability and apoptosis were both affected. Additionally, it was observed that the miR-128-3p level was decreased in LPS-induced hDPCs and was negatively correlated with the level of PVT1 in cells, suggesting the targeting relationship between PVT1 and miR-128-3p.

LncRNA has attracted extensive attention for its role in pathological reactions over the past few decades. PVT1 has been studied as an oncogene for several decades, and its carcinogenic effect has been proven in a variety of tumors, such as in lung cancer, colon cancer, hepatic carcinoma, and breast cancer.^5,26-28^ In recent years, the role of PVT1 in inflammatory diseases has become a research hotspot in related fields. For example, Meng et al^21^ reported that overexpression of PVT1 aggravates the LPS-induced osteoarthritis inflammation by regulating the HMGB1/TLR4 axis and NF-κB pathway. Hu et al^7^ demonstrated that PVT1 promotes the development of acute pancreatitis by inducing autophagy activation of pancreatic acinar cells by regulating mir-30A-5p/Beclin-1 axis. In the present study, the PVT1 level was found to be elevated in saliva of pulpitis patients compared with healthy patients, and subsequent ROC curve analysis confirmed the clinical diagnostic role of high PVT1 levels in pulpitis, suggesting PVT1 may be involved in the pathological mechanism of pulpitis. Our above results are consistent with the expression of PVT1 in pulpitis reported recently by Lei et al.^12^ LPS is an endotoxin derived from gram-negative bacteria and is often used to establish the in-vitro and in-vivo models of inflammation and infection.^24^ In this study, the in-vitro pulpitis cell model was successfully constructed by LPS stimulation on hDPCs, which was accompanied by suppression of cell proliferation, and acceleration of cell apoptosis and inflammatory reaction. We demonstrated in our model that inhibition of PVT1 alleviates LPS-induced cell damage. In addition, it was found that miR-128-3p was negatively regulated by PVT1 in hDPCs.

MiR-128-3p is one of the newly discovered miRNAs in recent years, and its role in tumors, neuropathic pain, inflammatory and other diseases has been confirmed.^16,25^ Cai et al^2^ showed that miR-128-3p induces anti-apoptotic efficacy by inhibiting the activation of caspase-3 in non-small–cell lung cancer cells. Yang et al^25^ reported that overexpression of miR-128-3p in septic cell models improves LPS-induced cell inflammation and apoptosis by inhibiting the Smad signaling pathway. miR-128-3p was one of the target genes of PVT1, and the target relationship of PVT1 and miR-128-3p was affirmed by luciferase reporter gene assay. These results showed that PVT1 inhibition correspondingly increased miR-128-3p expression and ameliorated LPS-induced hDPCs damage. Meanwhile, inhibition of miR-128-3p reversed the alleviating effect of PVT1 inhibition on LPS-induced cell damage. These results further verified the relationship between PVT1 and miR-128-3p.

The expression level of PVT1 in patients’ saliva was investigated. However, since we did not determine the PVT1 expression in dental pulp tissue, gingival crevicular fluid, or blood of patients, we could not determine whether the expression of PVT1 was consistent in these three clinical samples, which was a key limitation of this study. In future studies, the function of PVT1 and miR-128-3p in pulpitis needs to be further explored, and the sample size of the subject population must be increased to support the experimental results.

Studies have shown that patients with pulpitis often suffer from caries and some periodontal diseases. Another limitation of this study is that when patients with pulpitis are included in the case group, there is no strict distinction between pure pulpitis and periodontitis with pulpitis. This phenomenon may lead to increased inflammation in patients and high levels of inflammatory factors. Therefore, studies of pulpitis caused by caries and bacterial infection should be carried out.

In summary, this study preliminarily revealed that PVT1 plays a part in promoting the progression of pulpitis by inhibiting the proliferation of hDPCs and promoting the apoptosis and inflammatory response of hDPCs at the cellular level through targeting miR-128-3p.

## Conclusion

Our results suggested that PVT1 and miR-128-3p may be new targets for pulpitis control or treatment. However, the impact and mechanism of PVT1 and miR-128-3p have yet to be demonstrated in vivo.
